# Houshiheisan promotes angiogenesis via HIF-1α/VEGF and SDF-1/CXCR4 pathways: *in vivo* and *in vitro*

**DOI:** 10.1042/BSR20191006

**Published:** 2019-10-15

**Authors:** Yangyang Xiang, Xiaoquan Yao, Xuan Wang, Hui Zhao, Haiyan Zou, Lei Wang, Qiu-Xia Zhang

**Affiliations:** 1 School of Traditional Chinese Medicine, Capital Medical University, Beijing, China; 2 Beijing Key Lab of TCM Collateral Disease Theory Research, Beijing, China

**Keywords:** angiogenesis, cerebral ischemia, chemokine, HIF-1α/VEGF, Houshiheisan

## Abstract

*Rationale*: Houshiheisan (HSHS), a classic prescription in traditional Chinese medicine (TCM), has remarkable efficacy in the treatment of ischemic stroke.

*Objective*: To investigate the pro-angiogenic effect and molecular mechanism of HSHS for stroke recovery.

*Methods and results*: The rat permanent middle cerebral artery occlusion (pMCAO) model was constructed by suture method, HSHS (5.25 or 10.5 g/kg) and Ginaton (28 mg/kg) treatment was intragastrically administrated at 6 h after modeling which remained for 7 consecutive days. Pathological evaluation conducted by Hematoxylin–Eosin (HE) staining and the results showed that HSHS alleviated blood vessel edema, reduced the damage to blood vessels and neurons in the ischemic areas. Immunostaining, quantitative real-time fluorescence PCR results showed that HSHS up-regulated pro-angiogenic factors including platelet endothelial cell adhesion molecule-1 (cluster of differentiation 31 (CD31)), vascular endothelial growth factor (VEGF), vascular endothelial growth factor A (VEGFA), VEGF receptor 2 (VEGFR2), angiopoietin-1 (Ang-1), while down-regulated angiopoietin-2 (Ang-2), stromal cell derived factor-1 (SDF-1), and cxc chemokine receptor 4 (CXCR4) expression in infarct rat cortex, and similar results were obtained in subsequent Western blot experiment. Furthermore, CCK8 assay and transwell migration assay were performed to assess cell proliferation, migration, and tube formation. The medicated serum (MS) of HSHS appeared to have beneficial effects for immortalized human umbilical vein cells (Im-HUVECs) on proliferation and migration after persistence hypoxia. Western blot analysis revealed that the expression of hypoxia inducible factor-1α (HIF-1α), VEGFA, Ang-1, Ang-2, and CXCR4 were significantly up-regulated while Ang-2 was down-regulated by HSHS MS treatment compared with vehicle group *in vitro*.

*Conclusion:* The present study suggests a novel application of HSHS as an effective angiogenic formula for stroke recovery.

## Introduction

Ischemic cerebral stroke (ICS) is a worldwide disease characterized as high morbidity and mortality, which brings adverse outcomes such as the dysfunction in language, cognition, and motor [1]. Timely thrombolysis therapy brings great benefits to the patients with ICS, but it fails to effectively improve cerebral perfusion in those people [2]. Various sequelae such as hemiplegia, aphasia, and dysphagia still plague some patients which have received thrombolytic therapy [3]. In recent years, neuroprotectors perform pretty well in laboratory studies, however, they cannot replace thrombolytic therapy in current clinical applications. More effective treatments for ICS are still worth investigating.

Sufficient amount of evidence demonstrated that effective collateral circulation was an important compensatory mechanism after cerebral ischemia, which could protect brain tissue and improve prognosis by increasing blood perfusion in ischemic penumbra (IP) [4,5]. Angiogenesis plays a key role in the establishment of collateral circulation. Importantly, vascular endothelial growth factor (VEGF) is a molecular hub of angiogenesis, which can provide a favorable environment for angiogenesis by regulating the expression of multifarious angiogenic molecules [6]. When VEGF binds to its receptor VEGFR2 (VEGF receptor 2), a membrane protein in endothelial cells, it can promote cell proliferation, migration, and tube formation [7]. Furthermore, hypoxia inducible factor-1 (HIF-1) signaling can also participate in VEGF-mediated angiogenesis under hypoxia conditions. HIF-1 consists of two subunits: HIF-1α and HIF-1β. HIF-1α rapidly enters the nucleus to bind with HIF-1b, which up-regulates the expression of several angiogenic genes to promote forming new blood vessels under hypoxia environment [8].

The extension of blood vessels is another important manifestation in angiogenesis, in which chemokine family porteins play a prominent role during this process. Stromal cell derived factor-1 (SDF-1) is a representative of the chemokine family, which specifically binds to cxc Chemokine Receptor 4 (CXCR4) [9]. The SDF-1/CXCR4 pathway makes ischemic vessels extend toward the areas with adequate blood supply through guiding the migration and adhesion of endothelial cells [10]. It is worth pointing that SDF-1 is also a definitive target of HIF-1 signaling, involved in the post-ischemia angiogenesis [11].

Houshiheisan (HSHS) is a classic prescription for stroke in traditional Chinese medicine (TCM), which has been applied in clinical treatment safely and effectively for approximately 2000 years. At present, HSHS is mainly used in the treatment of ICS, headache, and hypertension, especially in acute stroke [12–15]. Our previous studies showed a couple of neuro-protective mechanism of HSHS such as increasing the secretion of brain-derived neurotrophic factor (BDNF), decreasing the accumulation of amyloid precursor protein (APP), and mediating axon growth via Nogo and Netrin signals [16,17]. However, the molecular mechanism of HSHS promoting angiogenesis after ischemia has not been fully elucidated. Therefore, in the present study, we tried to explore the molecular mechanism of HSHS in promoting angiogenesis via HIF-1α/VEGF and SDF-1/CXCR4 pathways.

## Materials and methods

### Reagents and antibodies

DMEM medium with 4.5 g/l glucose (10-013-CV, Corning, Corning, NY, U.S.A.), DMEM glucose-free medium (11966-025, Invitrogen, Waltham, MA, U.S.A.), CCK8 reagent (CK04, Dojindo, Kumamoto, Japan), penicillin–streptomycin (C0222, Beyotime, Shanghai, China), fetal bovine serum (FBS, 35-010-CV, Corning, Corning, NY, U.S.A.), Matrigel (356234, BD, Franklin Lakes, NJ, U.S.A.), and Triton X-100 (T8787, Merck Millipore, St. Louis, MO, U.S.A.), TRIzol (Invitrogen, Carlsbad, CA, U.S.A.), FastQuant RT Kit (with gDNase) (Tiangen, Beijing, China), SuperReal PreMix Plus SYBR Green kit (Tiangen, Beijing, China).

#### Primary antibodies

Cluster of differentiation 31 (CD31) (mouse monoclonal, Abcam, Cambridge, U.K.), SDF-1α (rabbit polyclonal, Abcam), CXCR4 (rabbit monoclonal, Abcam), vascular endothelial growth factor A (VEGFA) (mouse monoclonal, Abcam), HIF-1α (mouse monoclonal, Abcam), angiopoietin-1 (Ang-1) (rabbit polyclonal, Abcam), angiopoietin-2 (Ang-2) (rabbit polyclonal, Genetex, Irvine, CA, U.S.A.), and GAPDH (mouse monoclonal, Genetex).

#### Secondary antibodies

Goat anti-mouse and goat anti-rabbit IgG-FITC (SouthernBiotech, Birmingham, AL, U.S.A.). Horseradish peroxidase-linked goat-anti-rabbit and goat-anti-mouse IgG (Neobioscience Biotech Co., Ltd, Beijing, China).

### Preparation of HSHS

HSHS consists of the following 13 traditional Chinese herbs: *Flos Chrysanthemi, Radix Saposhnikoviae, Ramulus Cinnamomi, Rhizoma Chuanxiong, Radix et Rhizoma Asari, Radix Platycodonis, Rhizoma Atractylodis macrocephalae, Poria, Rhizoma Zingiberis, Radix Angelicae sinensis, Radix et Rhizoma Ginseng, Radix Scutellariae* and *Concha Ostreae.* All the herbs were purchased from Tong-ren-tang Chinese Medicine Co., Ltd. (Beijing, China) and authenticated by Associate Professor Jia Li (Capital Medical University, Beijing, China) according to the Chinese Pharmacopoeia (2015 edition). The proportion of each herb was prepared according to the instructions in *the Synopsis of Golden Chamber*. Drugs were prepared by following steps: (1) immerse the herbs in distilled water for 2 h; (2) after a brief boiling, stew with gentle heat for 1 h and then collect the decoction; (3) add moderate water and repeat the boiling process once, then combine and mix the decoction in (2) and (3); (4) transfer the decoction into a flat plate, and then put it above a 90°C water bath to volatilize until the decoction reaches a suitable volume. In the present study, two concentrations of HSHS extract were used, which respectively contained 1.05 or 0.525 g crude herbs in 1 ml. The chemical constituents in the extract were identified with high-performance liquid chromatography (HPLC) analysis and published in our previous paper [17].

### Animals and ethics

The study was approved by the Institution Animal Care and Use Committee of Capital Medical University [Ethical License No. AEEI-2016-054], and all the animal experimental manipulations were fully compliant with the Guidance Suggestions for the Care and Use of Laboratory Animals published by the Ministry of Science and Technology of China. Three-month-old healthy male Sprague–Dawley rats (SPF grade) were purchased from Beijing Vital River Laboratory Animal Technology Co.Ltd., China. All the rats were raised in a specific pathogen-free environment provided by the laboratory animal center of the Capital Medical University, China [Animal License No. SYXK (Jing) 2010-0020].

### Establishment of permanent middle cerebral artery occlusion model and drug administration

Fifty healthy rats were randomly divided into five experimental groups named sham group (*n*=10), permanent middle cerebral artery occlusion (pMCAO) group (*n*=10), HSHS 5.25 g/kg (*n*=10), HSHS 10.5 g/kg (*n*=10), and Ginaton 28 mg/kg group (*n*=10). The pMCAO model was constructed based on a standard method previously published [18]. Rats were calmly placed in a 2:1 N_2_O:O_2_ environment, then anesthetized using a face mask with 3% isoflurane properly. The rat was fixed in a supine position, the initial section of the right common carotid artery, internal carotid artery, and external carotid artery were carefully exposed by blunt dissection. Then an emboli made of 4-0 monofilament nylon thread was inserted from the internal carotid artery to block the blood perfusion of the area supplied by right middle cerebral artery. The same operation was performed on the rats in sham group without insertion of the nylon suture. Two rats in the pMCAO group and one in HSHS group died within 7 days.

The equivalent dose of HSHS in the present study was calculated with a body surface area normalization method according to the normal clinical dose [17]. The first-time administration was performed at 6 h after modeling using an intragastric method, and then it was administered every 24 h during 7 consecutive days. HSHS 5.25, HSHS 10.5 g/kg, and Ginaton 28 mg/kg groups were treated with the corresponding doses of drug. Sham group and pMCAO group were fed with normal saline at 1 ml per 100 g body weight.

### Pathological assessments

Three rats were randomly selected from each group to conduct pathological evaluation on day 7. The whole brain of the rats were carefully removed after a trans-cardiac perfusion with 0.9% normal saline and 0.1% M phosphate buffered solution (PBS, pH 7.4) containing 4% paraformaldehyde, respectively. The tissue was fixed with 4% paraformaldehyde and embedded in paraffin, then sliced into 3-μm thick sections for Hematoxylin–Eosin (HE) staining. The pathological changes in infarct cortex were observed through optical microscope (E100, Nikon, Tokyo, Japan), followed by a quantitative analysis of survival neurons and vascular lumens from five random views on each section.

### Immunohistochemistry staining

The paraffin-embedded sections were kept at 60°C for 24 h in the oven, then we proceeded in sequence with xylene deparaffinization, ethanol gradient (100–70%) hydration, and citric buffer (pH 6.0) antigen retrieval. A 10-min incubation with 3% hydrogen peroxide aiming to inactivate endogenous enzymes was performed in a wet box at room temperature. The sections were rinsed with phosphate-buffer saline (PBS) and incubated with the primary antibody (SDF-1 at 1:100 and CXCR4 at 1:80) overnight at 4°C. The next day, the slides were rinsed and incubated with the corresponding secondary antibody for 30  min followed by 3,3′-diaminobenzidine (DAB) and Hematoxylin staining, respectively. The slides were photographed using an Olympus fluorescence microscope (Tokyo, Japan) and a digital photo microscope (Leica, Wetzlar, Germany). NIS-Elements Basic Research image acquisition system (Nikon) was used for the positive area calculation in each section [19].

### Immunofluorescence staining

Brain samples were fixed with 4% paraformaldehyde overnight at 4°C, and sliced into 3-μm-thick sections. The sections were permeabilized with 0.1% Triton X-100 for 10 min, and then washed with PBS twice. Sections were incubated in a dark box with VEGFA primary antibodies (1:150) for 24 h at 4°C. After that, the sections were again washed with PBS twice, and incubated with appropriate fluorescence–conjugated secondary antibodies for 2 h at room temperature. DAPI reagent was used to stain the cell nucleus. All of the images were obtained using fluorescence camera microscopy (Leica, Wetzlar, Germany), and positive expression was quantified using the Image-Pro Plus software [20].

### Quantitative reverse-transcription polymerase chain reaction

The total RNA was collected using TRIzol reagent according to the manufacturer’s instructions. A total of 2 μl of total RNA extract was subjected for reverse transcription into cDNA using FastQuant RT Kit (with gDNase). Then the quantitative PCR was performed with SuperReal PreMix Plus SYBR Green kit and CFX Connect Optics Module (Bio-Rad, Hercules, CA, U.S.A.) [21]. The following primers were using in this assay:

VEGF: -F-5′-GCACGTTGGCTCACTTCCAG-3′,
-R-5′-TGGTCGGAACCAGAATCTTTATCTC-3′;
VEGFR2: -F-5′-GAGCATGTTGCCTGTCACGA-3′,
-R-5′-CGGACGGAATTTGATTAGGGTAG-3′;
Ang-1: -F-5′-ACCGTGAGGATGGAAGCCTAGA-3′,
-R-5′-AATGAACTCGTTCCCAAGCCAATA-3′;
Ang-2: -F-5′-CTTCAAGTCAGGACTCACCACCA-3′,
-R-5′-CCACCCATGTCCATGTCACAG-3′;
β-actin:-F-5′-GGAGATTACTGCCCTGGCTCCTA-3′,
-R-5′-GACTCATCGTACTCCTGCTTGCTG-3′

### Preparation of HSHS medicated serum

HSHS medicated serum (MS) was extracted according to a published method [22]. A total of 20 rats were randomly divided into control and HSHS 10.5 g/kg group (*n*=10), and anesthetized at 1 h after the last administration, and blood samples were taken from the aseptic abdominal aorta by vacuum blood collection tubes (BD, NJ, U.S.A.). The blood was first placed at room temperature for 2 h, then centrifuged at 3000rpm for 15 min. After incubating at 30-min water bath at 56°C, the serum was filtered with 0.22-μm microporous membrane and stored at −80°C after sterilization.

### Cell culture and oxygen-glucose deprivation

The immortalized human umbilical vein cells (Im-HUVECs) were donated by the Cancer Laboratory of Beijing Traditional Chinese Medicine Hospital (Beijing, China) and authenticated prior to use. Cells were cultured in a full DMEM culture medium supplemented with 10% (v/v) FBS and 1% (v/v) penicillin–streptomycin, and all the subsequent manipulations implemented after the Im-HUVECs grew to 85–95% area of the bottle. After 12 h of serum-starving, Im-HUVECs were cultured in glucose-free DMEM medium and exposed to oxygen-glucose deprivation (OGD) (environment <v/v>: 94% N_2_, 5% CO_2_, and 1% O_2_) for 6 h [23].

### Cell viability

Cells were divided into six groups: normoxia, hypoxia, 2.5% HSHS MS, 5% HSHS MS, 10% HSHS MS, and 20% HSHS MS, and cultured in 96-well plates at a density of 1 × 10^5^/ml. Viability evaluation was performed after 24 h MS treatment using CCK8 reagent according to the manufacturer’s instructions. After an appropriate time incubation at 37°C, the absorbance value (OD) was detected at 450 nm wavelength using a microplate reader. Cell viability was quantified with the following formula: 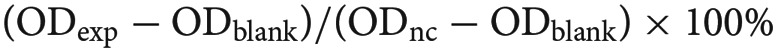 [24].

### Transwell migration assay

Im-HUVECs were resuspended in DMEM full medium containing 2.5% bovine serum albumin (BSA) at the density of 2.5 × 10^5^/ml, adding 200 μl suspension into each upper chamber of transwell inserts with 8.0-μm pore size polycarbonate membrane filters (#3422, Corning, Corning, NY, U.S.A.). Then the cells were exposed to OGD for 6 h, while normal control cells were cultured in normal oxygen environment for the same time. When the above manipulations had finished, 800 μl control (Corning DMEM containing 10% FBS) or treatment medium (Corning DMEM with 2.5, 5, 10, or 20% HSHS MS) was added to the lower chamber. After 12-h incubation, the non-migrating cells on the upper side of filters membrane were carefully removed with a cotton bud. The migrated cells on the lower side of the chamber filter were fixed with 4% paraformaldehyde in PBS and then stained with DAPI for 5 min. The migrated cells were quantified with the integrated optical density (IOD) from five different views in each membrane at 100× magnification (E100, Nikon, Tokyo, Japan) [25].

### Detection of HIF-1α, VEGFA, Ang-1, Ang-2, SDF-1, and CXCR4 protein expression

Ischemic brain protein samples for sodium dodecyl sulfate/polyacrylamide gel electrophoresis (SDS/PAGE) were prepared according to the method in the previous paper [17]. The collected Im-HUVECs from normoxia, vehicle, 2.5% MS, 5% MS, 10% MS, and 20% MS groups were lysed with RIPA for approximately 20 min on ice and centrifuged at 12000×***g***, 4°C for 15 min. The protein was quantified by BCA assay. Thirty micrograms of total protein from each sample was subjected to electrophoresis, and then the proteins were transferred on to PVDF membranes in electroacoustic buffer at 300 milliamperes for 45 min. The membranes were blocked with 5% nonfat milk dissolved in Tris-buffered saline with 0.1% Tween-20 (TBST) for 1 h, and incubated in primary antibodies (including VEGFA at 1:2000, Ang-1 at 1:4000, Ang-2 at 1:5000, SDF-1α at 1:1000, CXCR4 at 1:2000, and HIF-1α at 1:3000) overnight at 4°C. Next day, the membranes were washed in TBST for 5 × 5 min, incubated in appropriate secondary antibodies for 1 h, and then the membrane was washed for another 5 × 5 min at room temperature. Membranes were exposed and fixed after ECL liquid reaction, the density of each band was quantified by using National Institutes of Health (NIH) ImageJ analysis software. GAPDH was used as the loading control.

### Statistical analysis

Statistical analysis was conducted using SPSS 22.0 software. Normally distributed or approximately normally distributed data were presented as mean ± SD. One-way ANOVA was used for the comparison among multiple groups, and least significant difference (LSD) was used for post hoc comparisons between any two groups. The statistical significance was set at *P*<0.05.

## Results

### HSHS alleviates the injury of neurons and blood vessels after cerebral ischemia in rats

**Figure 1:**
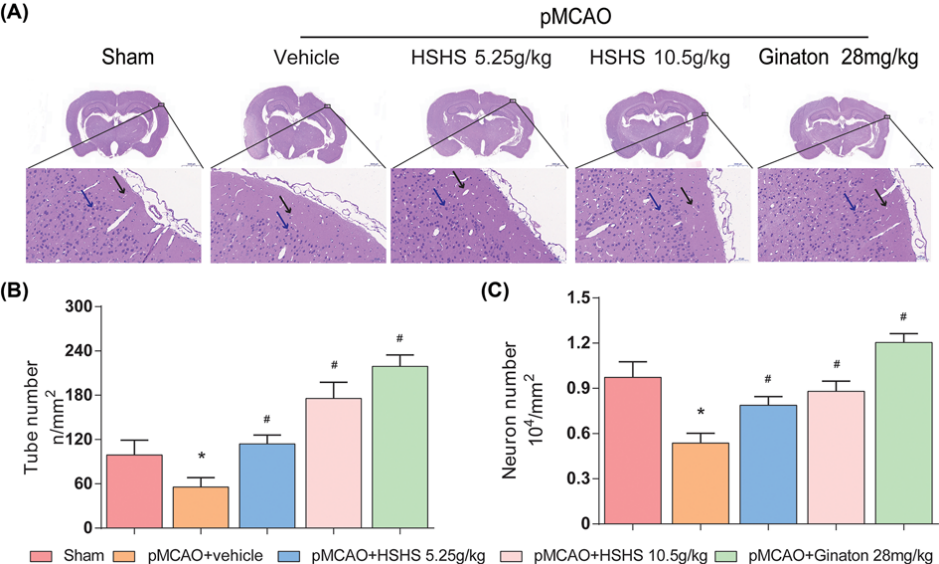
HSHS alleviates the injury of neurons and blood vessels after cerebral ischemia in rats (**A**) HE stained images for pathological assessment in different groups of rats were shown, black arrows indicate blood vessels and blue arrows indicate surviving neurons. Scale bar = 2000/50 μm. (**B,C**) Quantification analysis of surviving neuron number and tube number. *n*=4. Data are presented as mean ± SD. *P<0.01 vs. Sham, ^#^*P*<0.01 vs. pMCAO+vehicle.

HE staining was conducted for pathological assessment in this work. Different degree of lesion appeared in the right cerebral cortex after pMCAO modeling was observed by optical microscope (Figure 1A). The blood vessels in the infarcted cortex were malformed with obvious edema around the vessels. In addition, the morphology of vascular endothelial cells was altered and the intercellular connections were partially absent. Compared with the vehicle group, the ischemic injury in HSHS 5.25, 10.5 g/kg, and Ginaton group were significantly alleviated. Both the count of blood vessel lumen (*P*<0.01) and survival endothelial cells (*P*<0.01) in treatment groups increased than those in the model group (Figure 1B,C).

### HSHS promotes angiogenesis after cerebral ischemia in MCAO rats

**Figure 2:**
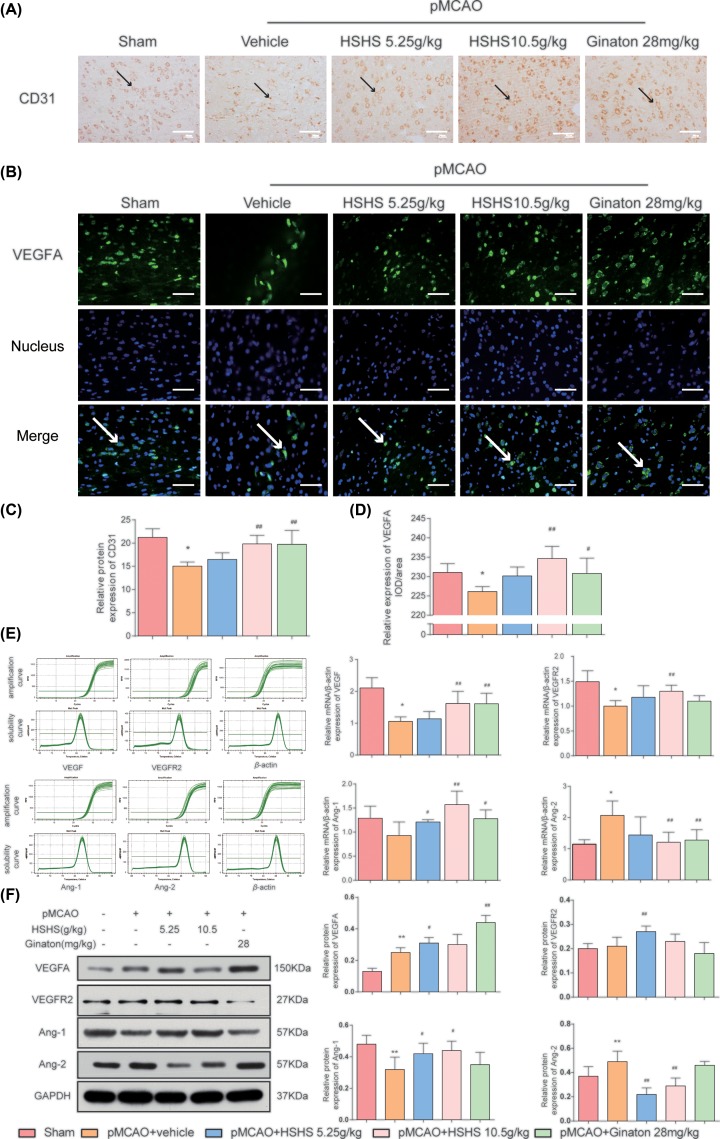
HSHS promotes angiogenesis after cerebral ischemia in MCAO rats (**A**) Representative photographs of immunohistochemistry staining of CD31 (indicated by black arrows) in different groups of rats. Scale bar = 50 μm. (**B**) Images of VEGFA immunofluorescence staining of the ischemic area of the cortex in different groups (indicated by white arrows). Scale bar = 200 μm. (**C,D**) Quantification analysis of relative protein expression of CD31 and VEGF. *n*=5. (**E**) Solubility curves, amplification curves, and quantitative PCR analysis of VEGF, VEGFR2, Ang-1, and Ang-2 expression. (**F**) Western blotting results for relative protein expression of VEGFA, VEGFR2, Ang-1, and Ang-2. *n*=5. Data are presented as mean ± SD. *P<0.01 vs. Sham, ^#^*P*<0.05 vs. pMCAO+vehicle, ^##^*P*<0.01 vs. pMCAO+vehicle. Abbreviation: PECAM-1, platelet endothelial cell adhesion molecule.

Expression of CD31 was detected by immunohistochemical staining to evaluate the effect of HSHS on angiogenesis *in vivo* (Figure 2A). The data showed that the positive expression of CD31 in the infarct cortex in vehicle group decreased compared with sham control (*P*<0.01), while HSHS 10.5 g/kg and Ginaton treatment up-regulated CD31 expression obviously compared with vehicle group (*P*<0.01) (Figure 2C). A significant reduction in VEGFA IOD (*P*<0.01) occurred after pMCAO, while HSHS 10.5 g/kg and Ginaton 28 mg/kg treatment significantly up-regulate VEGFA expression (*P*<0.01–0.05) (Figure 2B,D). Quantitative RT-PCR and Western blot were performed to detect the expression of multiple angiogenic molecules aiming to investigate the mechanism of HSHS on angiogenesis. Cerebral ischemia reduced the expression of VEGF (*P*<0.01), VEGFR2 (*P*<0.01), and Ang-1 (*P*<0.05) while increased Ang-2 (*P*<0.01) expression in the infarct rat cortex compared with the sham group. In comparison with vehicle group, HSHS 10.5 g/kg treatment up-regulated the expression of VEGF, VEGFR2, and Ang-1 and down-regulated Ang-2 expression (*P*<0.01–0.05). However, only HSHS 5.25 g/kg had obvious impact on Ang-1 expression compared with vehicle group (*P*<0.05) (Figure 2E). Interestingly, the expression of VEGFA of vehicle group was increased than that in sham group (*P*<0.01) in Western blot assay, and further up-regulated after HSHS treatment (*P*<0.01–0.05). No significant difference had been observed in the expression of VEGFR2 between sham group and vehicle group, suggesting that VEGFR2 might not be sensitive to hypoxia. The expression of Ang-1 and Ang-2 detected by immunoblotting were almost consistent with the results in PCR assay (*P*<0.01–0.05) (Figure 2F).

### HSHS promotes SDF-1/CXCR4 in rats ischemic cortex

**Figure 3:**
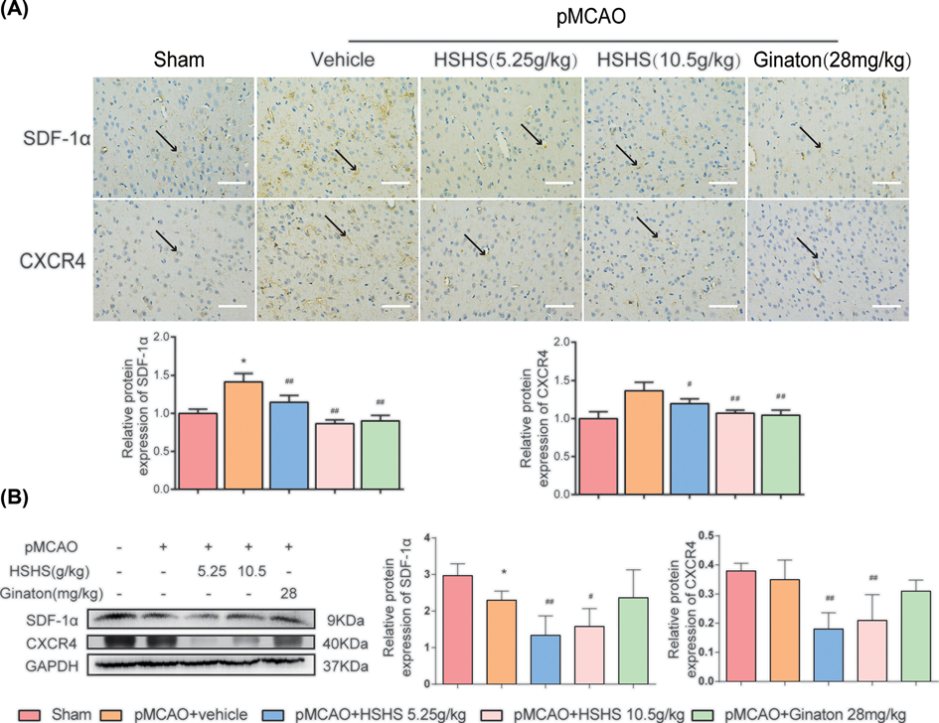
HSHS promotes SDF-1/CXCR4 in rats ischemic cortex **(A)** Representative photographs of immunohistochemistry staining of SDF-1α and CXCR4 (indicated by black arrows) in different groups of rats. Scale bar = 200 μm. **(B)** Quantification analysis of relative protein expression of SDF-1α and CXCR4. *n*=5. Data are presented as mean ± SD. *P<0.01 vs. Sham, ^#^*P*<0.05 vs. pMCAO+vehicle, ^##^*P*<0.01 vs. pMCAO+vehicle.

SDF-1α/CXCR4 pathway is considered as an upstream switch for many proliferation and migration pathways. The results of immunohistochemistry staining showed that pMCAO injury led to an increase in SDF-1α (*P*<0.01) and CXCR4 expression (*P*<0.01), whereas HSHS 5.25 g/kg, HSHS 10.5 g/kg and Ginaton treatment significantly could down-regulate the expression of both SDF-1α and CXCR4 (*P*<0.01–0.05) (Figure 3A). However, the data of Western blot were not totally consistent with that in immunostaining. The expression of SDF-1α expression in vehicle group was decreased than sham group (*P*<0.01), while the expression of CXCR4 in the two groups was not significant. The expression of both SDF-1α and CXCR4 could be down-regulated by HSHS after pMCAO (*P*<0.01–0.05) (Figure 3B).

### HSHS MS enhances endothelial cell viability after hypoxia *in vitro*

**Figure 4:**
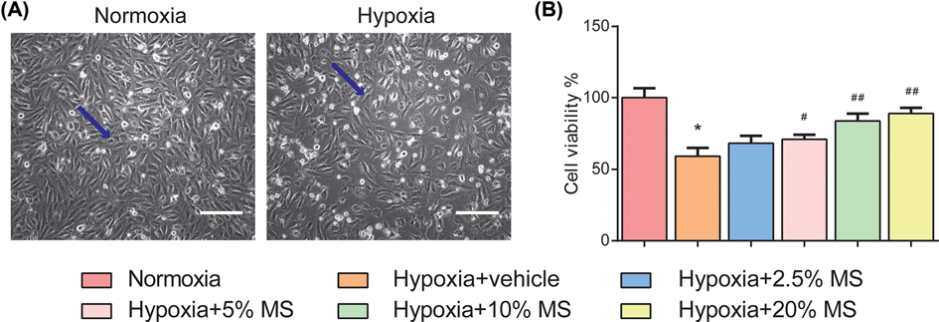
HSHS MS enhances endothelial cell viability after hypoxia *in vitro* (**A**) Representative images of normoxia and HUVECs after hypoxia (indicated by blue arrows). Scale bar = 300 μm. (**B**) The data of CCK8 cell viability assay are as follows. *n*=3. Data are presented as mean ± SD. *P<0.01 vs. Normoxia, ^#^*P*<0.05 vs. Hypoxia+vehicle, ^##^*P*<0.01 vs. Hypoxia+vehicle.

We have a lot of interesting findings in *in vitro* experiments as well. The data of CCK8 cell viability assay showed that 6-h OGD caused significant damage to the Im-HUVECs and caused their viability decreased in comparison with normoxia group (*P*<0.01). A total of 5, 10, and 20% HSHS MS treatment notably increased cell viability compared with hypoxia group (Figure 4). The results indicated that HSHS MS increased the viability and promoted the proliferation of endothelial cells after hypoxic injury.

### HSHS MS promotes endothelial cell migration

**Figure 5:**
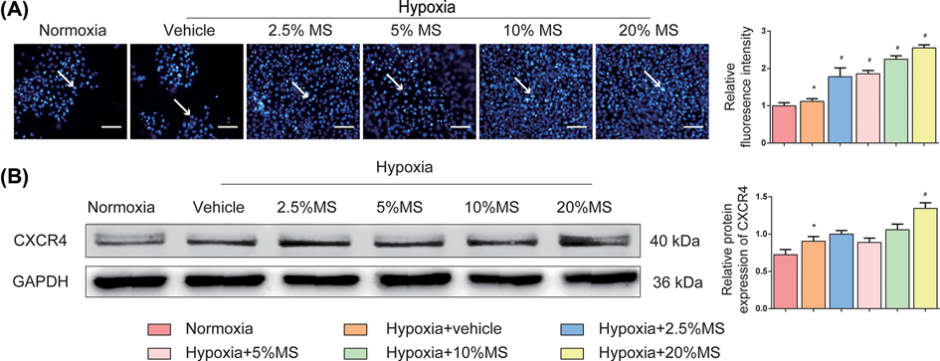
HSHS MS promotes endothelial cell migration *in vitro* (**A**) Representative images for transwell migration assay of vascular endothelial cells (indicated by white arrows), and quantitative results. Scale bar = 300 μm. *n*=5. (**B**) Western blotting results for CXCR4 and quantitative results of relative protein expression of CXCR4 to GAPDH. *n*=3. Data are presented as mean ± SD. *P<0.05 vs. Normoxia, ^#^*P*<0.01 vs. Hypoxia+vehicle.

Migration of vascular endothelial cells facilitates the formation of new blood vessels. The result of transwell migration assay showed that hypoxia stimulate caused an increase in migrated cells (*P*<0.05), and more cells migrated after 12-h HSHS MS treatment (*P*<0.01) (Figure 5A). Moreover,the expression of CXCR4 is increased in comparison with normoxia group after 6 h OGD (*P*<0.05), while only 20% HSHS MS further up-regulated the expression of CXCR4 in comparison with vehicle group (*P*<0.01) (Figure 5B).

### HSHS MS induces the activation of the pro-angiogenic factors in Im-HUVECs after hypoxia

**Figure 6:**
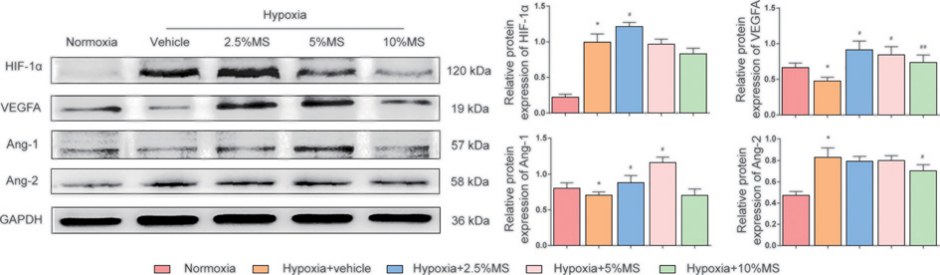
Western blotting results for HIF-1α, VEGFA, Ang-1, Ang-2, and GAPDH Quantitative results of Western blotting for HIF-1α, VEGFA, Ang-1, Ang-2 relative to GAPDH. *n*=3–5 (3 for Ang-1 and 5 for others). Data are presented as mean ± SD. *P<0.05 vs. Normoxia, ^#^*P*<0.05 vs. Hypoxia+vehicle, ^##^*P*<0.01 vs. Hypoxia+vehicle.

OGD leads an increase in HIF-1α (*P*<0.01) and Ang-2 expression (*P*<0.01), and a decrease in VEGFA (*P*<0.01) and Ang-1(*P*<0.05). In comparison with the vehicle group, 2.5% HSHS MS up-regulated the expression of HIF-1α (*P*<0.05); 2.5 and 5% HSHS MS up-regulated the expression of VEGFA and Ang-1 (*P*<0.01); only 10% HSHS MS treatment down-regulated the expression of Ang-2 (*P*<0.05) (Figure 6).

## Discussion

The damage in ICS mainly derives from the persistent hypoxia induced by insufficient blood perfusion, while collateral circulation is well known as an important protection and compensation mechanism which can increase the blood perfusion affecting the prognosis of ICS [26]. Angiogenesis is the later stage of collateral circulation establishment, which brings beneficial outcomes to ICS, such as reducing brain tissue damage and maintaining neurological function [27]. This study confirms that HSHS promotes angiogenesis, protects blood vessels and neurons after cerebral ischemia. The pro-angiogenic effects may relate to the regulation of HSHS on pro-angiogenic factors such as VEGF, Ang-1, Ang-2 and the chemokines.

Our previous study showed that the major five chemical components in HSHS extract were chlorogenic acid, luteolin-7-O-glucoside, 3,5-di-caffeoylquinic acid, apigenin-7-O-glucoside, and 4,5-di-caffeoylquinic acid [17]. These substances have neuroprotective functions such as anti-inflammatory, anti-apoptotic, and anti-free radical damage. Especially, apigenin, the aglycone of apigenin-7-O-glucoside, has been shown to have a clear pro-angiogenic effect [28].

As mentioned above, effective angiogenesis can reduce brain damage by increasing blood perfusion. In the present study, the results of HE staining showed that HSHS significantly alleviated the damage in infarct cortex tissue, increased the counts of survival neurons and blood vessels of pMCAO rats. All of these provided solid evidence to support that HSHS has protective effect on neurons and blood vessels after cerebral ischemia [29]. Thus, we detected the expression of CD31 to verify the pro-angiogenic effects of HSHS. It has been well documented that CD31 which is widely used to assess angiogenesis, the highly expressed CD31 indicates active proliferation of endothelial cells. Our data showed that HSHS obviously increased the expression of CD31 after pMCAO, suggesting that HSHS promoted endothelial cells proliferation and angiogenesis in infarct brain.

Endothelial cells are mainly involved in two stages of angiogenesis: proliferate to form new blood vessels, and migrate to prolong blood vessels and form an anastomosis with perfused blood vessels [30]. Thus, we highlight the role of HSHS in angiogenesis on cell proliferation, migration, and tube formation *in vitro*. The result of CCK8 assay showed that HSHS MS significantly enhanced cell viability of HUVECs, indicating that HSHS facilitated endothelial cell mitosis and proliferation after hypoxic injury. Hypoxia causes a spontaneous endothelial cell migration, and the amount of migrated cells can be further expanded by HSHS treatment. Importantly, we also observed that low concentration HSHS MS treatment could significantly increase the count of capillary-like structure which was formed by endothelial cells after persistent hypoxia in preliminary experiments. These results demonstrated that HSHS promoted endothelial cells to form new blood vessels by enhancing cell proliferation and migration.

The regulation of pro-angiogenic factors has influence on angiogenesis. VEGF, a heparin binding growth factor discovered in 1989, specifically promotes the mitosis of vascular endothelial cells [31]. The exons of VEGF gene can be spliced into several mRNA fragments to express more than five different isomers, which are collectively known as VEGFA [32]. The binding of VEGF and VEGFR2 activates multiple angiogenic signals, of which the angiopoietin pathway is the most important one [33]. In our study, pMCAO operation led to a decrease in VEGF, VEGFR2, Ang-1, and an increase in Ang-2 expression, while HSHS up-regulated those pro-angiogenic factors but down-regulated Ang-2. Ang-1 and Ang-2 play different roles in the process of angiogenesis [34]. The high expression of Ang-2 in the early stage of angiogenesis causes endothelial destabilization, whereas Ang-1 highly expressed during the later stage of angiogenesis promotes the maturation of endothelial cells and improves the stability of neovascularization [35,36].

More important, mass of evidence demonstrated the positive effects of SDF-1/CXCR4 pathway on promoting the proliferation and migration of endothelial cells in angiogenesis [37]. SDF-1 is a representative small molecule chemokine to enhance cell migration capacity, which can rapidly increase the expression of F-actin protein and quickly change its distribution in cells [38,39]. CXCR4 is well known as the specific receptor of SDF-1, and highly expressed in vascular endothelial cells [40]. SDF-1 and CXCR4 are highly expressed in the early stage of angiogenesis, promoting endothelial cells migration to form blood vessels, and then down-regulate after new blood vessels are formed to stabilize the lumem structures [41,42]. Our data showed that HSHS significantly down-regulated the two proteins in ischemic brain tissue 7 days after pMCAO. Interestingly, the expression changes of SDF-1α and CXCR4 detected by immunostaining and WB assay was not identical. We thought that the influence of SDF-1α on cell migration after ischemia may only exist in the limited regions but not the whole ischemic brain cortex.

*In vitro*, we detected the expression of HIF-1α on OGD Im-HUVECs. HIF-1α is a heterologous transcription factor in the nucleus and highly sensitive to the concentration of oxygen [43]. Once hypoxic stimulus occurs, HIF-1α enters the nucleus and then forms a dimer with HIF-1β to up-regulate the transcription level of various pro-angiogenic molecular genes. The binding site of VEGF and HIF-1α locates at the 5′ end of VEGF gene called the hypoxic response element (HRE), therefore HIF-1α plays an important role in the angiogenesis regulated by VEGF under hypoxia situation [44]. In this study, the expression of HIF-1α spontaneously increased after OGD, and was further up-regulated by HSHS MS administration. Furthermore, we observed that OGD injury decreased VEGFA, Ang-1 expression but increased Ang-2 expression in OGD HUVECs, while HSHS MS treatment increased VEGFA, Ang-1 and decreased Ang-2 expression compared with the vehicle group. The results suggested that HSHS could initiate angiogenesis molecular regulation and promote endothelial cell formation stable blood vessels. In addition, we found HSHS MS treatment could up-regulate the expression of cellular CXCR4 after hypoxia, which further proved the pro-angiogenic effects of HSHS.

In conclusion, HSHS could protect neurons and endothelial cells in the ischemic areas after cerebral ischemia. And we infer that the mechanism of HSHS may derive from: (1) regulating the pro-angiogenic pathway HIF-1α/VEGF and Ang-1/Ang-2 to initiate and promote the angiogenesis process after hypoxia; (2) regulating SDF-1/CXCR4 pathway to activate and promote endothelial cell migration.

## Abbreviations

Ang-1: angiopoietin-1
Ang-2: angiopoietin-2
CCK8: cell-counting kit-8
cDNA: complementary DNA
CD31: cluster of differentiation 31
CXCR4: cxc chemokine receptor 4
FBS: fetal bovine serum
GAPDH: glyceraldehyde-3-phosphate dehydrogenase
gDNase: genomic deoxyribonuclease
HE: Hematoxylin–Eosin
HIF-1α: hypoxia inducible factor-1α
HSHS: Houshiheisan
ICS: ischemic cerebral stroke
Im-HUVEC: immortalized human umbilical vein cell
IOD: integrated optical density
MS: medicated serum
OD: optical density
OGD: oxygen-glucose deprivation
PBS: phosphate-buffered solution/phosphate-buffer saline
pMCAO: permanent middle cerebral artery occlusion
RIPA: radio immunoprecipitation assay
SDF-1: stromal cell derived factor-1
SPF: specific pathogen free
TBST: Tris-buffered saline with 0.1% Tween-20
VEGF: vascular endothelial growth factor
VEGFA: vascular endothelial growth factor A
VEGFR2: VEGF receptor 2
WB: westernblot
